# Impact of neoadjuvant chemotherapy with PELF-protocoll versus surgery alone in the treatment of advanced gastric carcinoma

**DOI:** 10.1186/1471-2482-14-5

**Published:** 2014-01-24

**Authors:** Catharina Ruf, Oliver Thomusch, Matthias Goos, Frank Makowiec, Gerald Illerhaus, Guenther Ruf

**Affiliations:** 1 Department of Surgery, University of Freiburg, Universitätsklinikum, Hugstetterstr. 55, D-79106 Freiburg, Germany; 2 Department of Oncology, University of Freiburg, Freiburg, Germany

**Keywords:** Neoadjuvant chemotherapy, PELF, Advanced gastric cancer

## Abstract

**Background:**

In a retrospective study we analyzed the impact of neoadjuvant chemotherapy (CTx) with the PELF - protocol (Cisplatin, Epirubicin, Leukovorin, 5-Fluoruracil) on mortality, recurrence and prognosis of patients with advanced gastric carcinoma, UICC stages Ib-III.

**Methods:**

64 patients were included. 26 patients received neoadjuvant CTx followed by surgical resection, 38 received surgical resection only. Tumor staging was performed by endoscopy, endosonography, computed tomography and laparoscopy. Patients staged Ib – III received two cycles of CTx according to the PELF-protocol. Adjuvant chemotherapy was not performed at all.

**Results:**

Complete (CR) or partial response (PR) was seen in 20 patients (77%), 19% showing CR and 58% PR. No benefit was observed in 6 patients (23%). Two of these 6 patients displayed tumor progression during CTx. Major toxicity was defined as grade 3 to 4 neutropenia or gastrointestinal side effects. One patient died under CTx because of neutropenia and was excluded from the overall patient collective. The curative resection rate was 77% after CTx and 74% after surgery only. The perioperative morbidity rate after CTx was 39% versus 66% after resection only. Recurrence rate after CTx was 38% and 61% after surgery alone; we detected an effective reduction of locoregional recurrence (12% vs. 26%). The overall survival was 38% after CTx and 42% after resection only. The 5-year survival rates were 45% in responders, 20% in non - responders and 42% in only resected patients. A subgroup analysis indicates that responders with stage III tumors may benefit with respect to their 5-year survival in comparable patients without neoadjuvant CTx. As to be expected, non-responders with stage III tumors did not benefit with respect to their survival. The 5-year-survival was approximated using a Kaplan-Meier curve and compared using a log-rank test.

**Conclusion:**

In patients with advanced gastric carcinoma, neoadjuvant CTx with the PELF- protocol significantly reduces the recurrence rate, especially locoregionally, compared to surgery alone. In our study, there was no overall survival benefit after a 5-year follow-up period. Alone a subgroup of patients with stage III tumors appear to benefit significantly in the long term from neoadjuvant CTx.

## Background

Gastric carcinoma is the second most common GI-cancer with a poor overall prognosis [1]. Surgical resection is the only curative treatment option in afflicted patients. Detrimentally, the overall resection rate is as low as 33%, and in less than 60% of these patients R0-resection is possible [2,3]. At the time of diagnosis, half of patients suffer from advanced tumor disease making curative resection uncertain at best [1,2]. The probability of lymph node metastasis rapidly increases with the depth of infiltration. Already patients with stage Ib tumors have a high likelihood of lymph node metastases and therefore have a high recurrence rate of up to 69%, even following curative surgery [1,2,4]. Locoregional recurrence is most common (87%), but peritoneal and liver metastasis occur as well (13%) [5,6]. These data dramatically illustrate the importance of detecting gastric carcinoma at earlier stages.

The current survival rate across all tumor stages ranges between 40% and 50% and is still achieved primarily by curative surgical resection [7]. Only patients with Ia-tumors have a reasonably good prognosis with a 5-year survival rate of 83%. The survival rates of patients with more advanced tumors quickly decreases to 69% in patients with Ib tumors, 43% in stage II, 28% in stage III and 8.7% in stage IV patients [8-15].

Perioperative chemotherapy was thought to improve this dire prognosis, especially in patients with advanced tumor stages (UICC Ib-III) by down-staging the tumor and increasing the rate of curative resection [1,2,16]. Intraoperative radiation or adjuvant radio-/chemotherapy with various regimens do in fact successfully reduce locoregional recurrence, but fail to improve the long-term outcome [5,11-15].

Neoadjuvant chemotherapy (CTx) on the other hand is believed to reduce intraoperative tumor cell dissemination as well as occult micrometastases. Strategically, it is hoped that this may increase the curative resection rate and reduce locoregional recurrence, thus improving the prognosis of advanced gastric carcinoma [2,7,17].

## Methods

From January 2000 to December 2006, we treated a total of 124 patients with gastric carcinoma. All patients with an early tumor stage (UICC Ia), distant metastases (liver or peritoneum), oesophageal tumor localization, concomitant active malignant disease or poor liver and kidney functions and patients who denied CTx were excluded from our study. 64 patients with stage Ib – III gastric carcinoma were included. They received either a combined modality treatment with neoadjuvant CTx and surgery or surgical resection only. All patients chose chemotherapy after informed consent. All data were assessed retrospectively.

Staging was performed using the AJCC/UICC classification by means of endoscopy, endoscopic sonography, computed tomography and laparoscopy. The standard surgical procedure consisted of total gastrectomy and entailed D-2 lymphadenectomy. Subtotal gastrectomy and D2-lymphadenectomy were restricted to early stages of intestinal-type cancers of the distal third of the stomach. The resection margins were examined by frozen sections intraoperatively. Neoadjuvant CTx was administered according to the PELF-protocol in two cycles at 0 and 6 weeks: Cisplatin 40 mg/m^2^, Epirubicin 35 mg/m^2^, Leucovorin 500 mg/m^2^ and 5-Fluoruracil 500 mg/m^2^. Chemotherapy was terminated in cases of gastrointestinal and grade 2 to 4 hematologic toxicities. Subsequent to CTx, a second CT-scan, endoscopy and endoscopic sonography were performed to restage the tumor. Objective response was evaluated by histological examination and classified as “complete”, “partial” or “no response”. Histological regression was graded as “little”, “moderate” or “strong”. Both groups were compared with respect to age, sex, symptoms, diagnosis, tumor localization (proximal/distal), histology (Lauren type and WHO classification), grading (low, medium, high), extent of surgery (total/subtotal gastrectomy), and resection status (R0/R1). Endpoint criteria were complications during therapy, tumor recurrence and survival. Furthermore, responders and non-responders were compared with regard to completion of CTx, distribution of tumor stages, resection status and long-term survival (5-year follow-up). The 5-year-survival was approximated using a Kaplan-Meier curve and compared using a log-rank test. Clinical follow-up spanned the time from initial diagnosis of gastric cancer until the last registered visit in the clinical records of our ‘Comprehensive Cancer Care Center Freiburg’ or the day of death. All cases of postoperative death, including patients who died of other causes, were included in the survival analysis.

All statistical calculations were performed using SPSS 15 (SPSS Inc., Chicago) software. Statistical analysis was performed using X test, t test, and Fisher’s exact test. Results were considered statistically significant at P < 0.05.

## Results

One Patient, who died under neoadjuvant chemotherapy, was excluded. In total, 64 patients with advanced gastric carcinoma staged UICC Ib-III were included in the study. 26 of these received preoperative CTx followed by surgical resection. The other 38 patients received resection only. Patient characteristics were well balanced in both groups (Table 1). Median age was 67.86 years. There were 21 women and 43 men. Stage Ib was diagnosed in 12 patients (19%), stage II in 20 (31%) and stage III in 32 (50%). The tumor was located in the proximal stomach in 46 patients. Most frequently, an poorly differentiated (G3) intestinal Lauren type (36/64) tumor was found. All 64 patients underwent surgical Resection. 56 patients received total gastrectomy with D2-lymphadenectomy, 8 patients received subtotal gastrectomy in cases of early tumor stages and intestinal-type tumors with distal localization. The overall curative resection rate (R0) was 75% (n = 48/64). Thus by definition, 16 patients received not curative resection (R1).

**Table 1:** Patients characteristics (n = 64)

	**Neoadjuvant CTx**	**Surgery n**
	**(n = 26/64)**	**(n = 38/64)**
Age		
Median (years)	64.5	73
(range)	(38–76)	(43–91)
Sex		
Males	20	23
Females	6	15
Tumor localization		
Proximal	20	26
Distal	6	12
Surgical procedure		
Total gastrectomy	25	31
Subtotal gastrectomy	1	7
D2-lymphadenectomy	26	38
Resection		
Curative (R0)	20	28
Palliative (R1)	6	10
UICC stage, pretherapeutic		
Ib	2	10
II	7	13
III	17	15
WHO classification		
Adenocarcinoma	20	31
Signet-cell carcinoma	6	7
Grading		
G 1-2	6	21
G 3	20	17
Lauren-classification		
Intestinal	15	21
Diffuse	11	17

Preoperative CTx was administered in 26 and completed in 18 patients. In 8 cases, CTx was aborted due to gastrointestinal toxicity, i.e. nausea, vomiting and diarrhea (n = 3/26), hematologic toxicity, i.e. neutropenia (n = 2/26), complete early tumor regression in one case and tumor progression in 2 further patients. We observed a histological response rate of 77% (n = 20/26), 5 patients showing complete and 15 patients displaying partial response. No histological benefit was detected in 6 patients (23%). In respect of the regression grade 75% showed submucosal subtotal cicatrisation, which complies with regressiongrade 2; the examination of the resected tissue of the remaining patients showed complete necrosis. (Regression grade was examined by JRSGC). The histological response was particularly significant among patients staged UICC III. Table 2 illustrates pre- versus postoperative tumor staging in both groups. The curative resection rate after neoadjuvant CTx was 77% (n = 20/26) and was accomplished in 17 responders and 3 non-responders. R1-resection was diagnosed in 6 cases (3 responders and 3 non-responders).

**Table 2:** Comparison of tumor stage distribution before and after chemotherapy (downstaging) and resection only

**UICC**	**Neoadjuvant CTx (n = 26/64)**		**Surgery (n = 38/64)**	
	**Before CTx**	**Postoperative**	**Preoperative**	**Postoperative**
Ib	2	10	10	16
II	7	7	13	9
III	17	3	15	7
IV	0	6	0	6

Postoperative complications developed in 35 of 64 patients. The complication rate was 39% in the group with chemotherapy (n = 10/26) and 66% after surgical resection only (n = 25/38). Common complications like pneumonia (7/64), pulmonary embolism (2/64) and urinary infection (1/64) occurred more frequently in the group without neodjuvant CTx. Complication like deep vein thrombosis appeared in both groups with the same frequency (2/64). Between the two groups, there was no difference with respect to the surgical complication rate, which arose in 8 of 26 patients subsequent to CTx and 15 of 38 patients after resection only. Anastomotic leakage occurred in 12% after CTx and 11% after resection only. Also intestinal obstruction (3/64), delayed nourishment (5/64) and wound infection (8/64) weren’t more frequent in the group with CTx (Table 3).

**Table 3:** Complications

	**Neoadjuvant CTx (n = 26/64)**	**Surgery (n = 38/64)**
Anastomotic leakage	3	4
Delayed nourishment	0	5
Intestinal obstruction	2	1
Wound infection	3	5
Pneumonia	1	6
Urinary infection	0	1
Lung embolism	0	2
Deep vein thrombosis	1	1

Overall recurrence rate was 52% (n = 33/64). CTx reduced the overall recurrence rate and was 39% in the neoadjuvant group versus 63% in the only surgery group (Table 4). Especially locoregional recurrence was reduced after chemotherapy and occurred in 12% versus 26% after resection only. Lymph node metastases merely occurred in only resected patients. However, peritoneal recurrence or distant metastases couldn’t be prevented by CTx.

**Table 4:** Localization of recurrence

	**Neoadjuvant CTx (n = 26/64)**	**Surgery (n = 38/64)**
Local	3	10
Peritoneal	3	4
Lymph node metastases	0	5
Distant metastases	4	5

**Figure 1:**
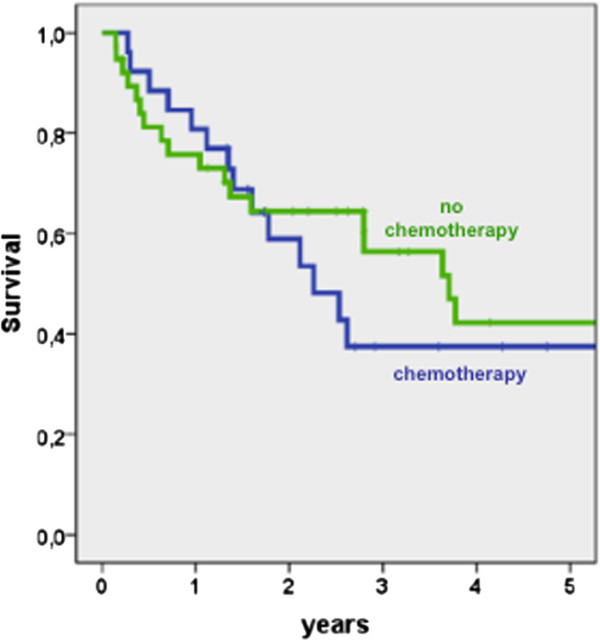
Survival of patients with preoperative chemotherapy versus surgery alone (Log rank p = 0,580).

**Figure 2:**
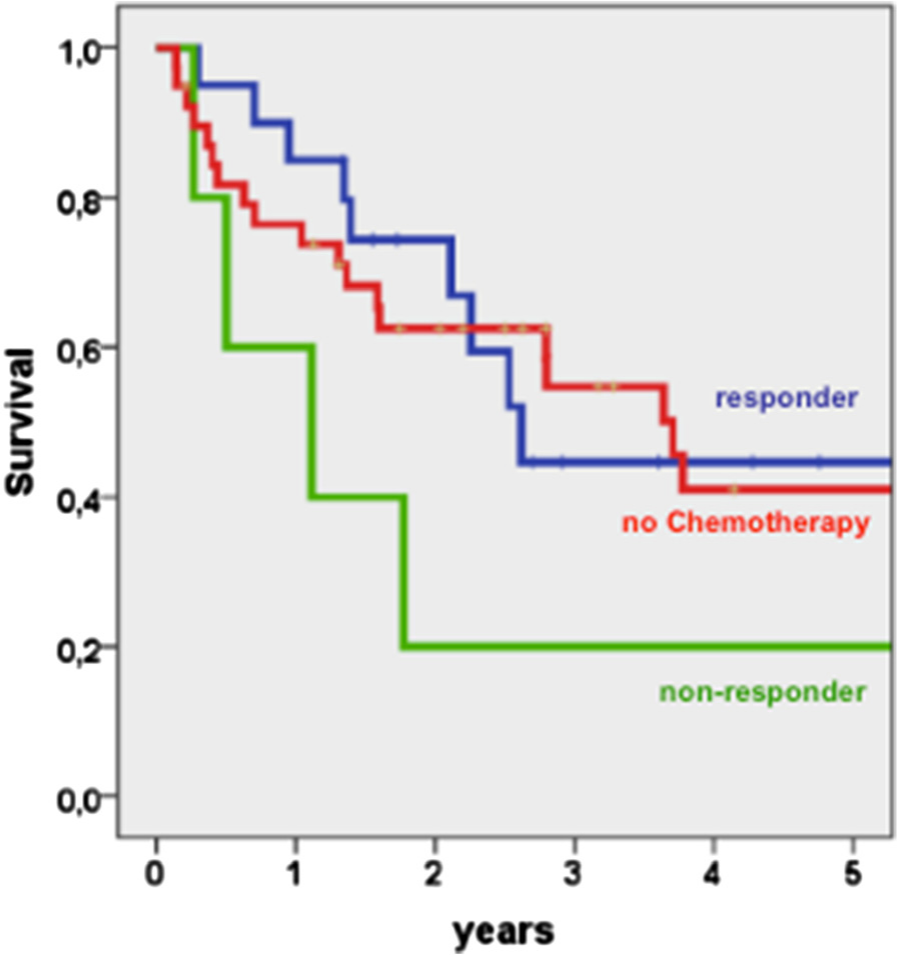
Survival of responders, non-responders and only resected patients (Log rank p = 0,283).

The overall survival was not improved by CTx (Figure 1). The 5-year survival rate was 38% after CTx versus 42% after surgery only. Not even a subgroup analysis comparing responders with only resected patients showed a benefit of CTx. The 5-year survival rate of responders was 44% versus the above mentioned 42% after surgery only. Non-responders to CTx had a worse survival rate of 20% (Figure 2).

**Figure 3:**
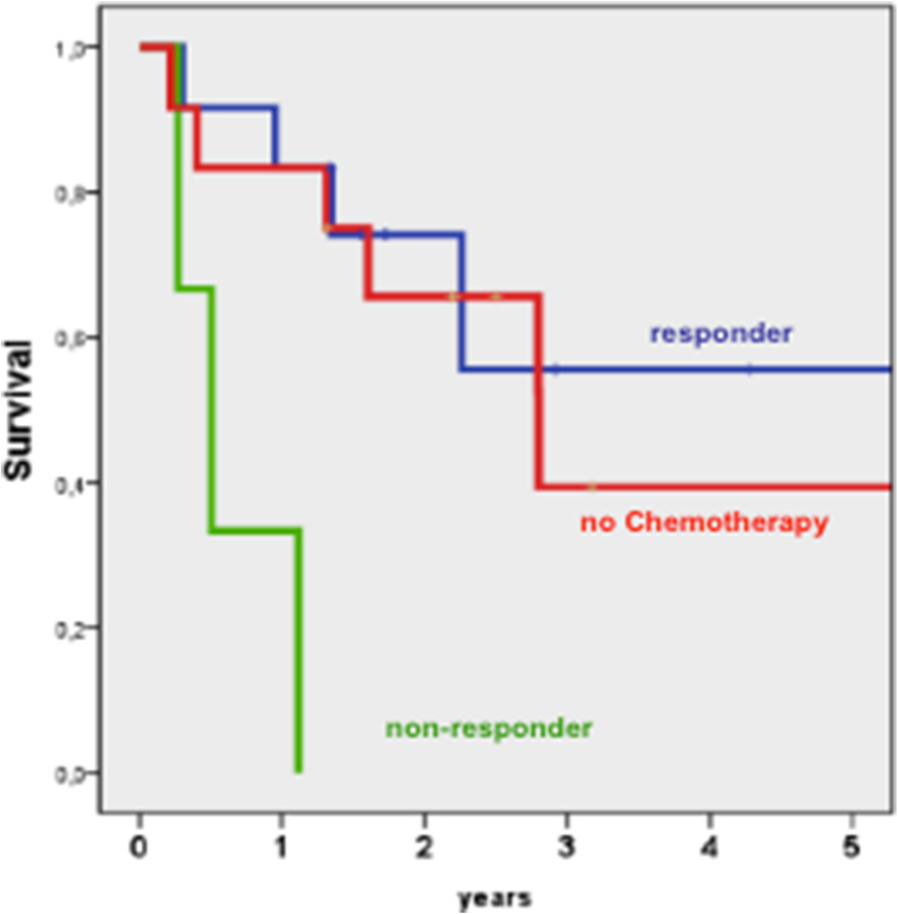
Survival curves of responders, patients without neoadjuvant chemotherapy and non-responders to neoadjuvant chemotherapy with stage III UICC only (log rank p = 0,002).

A subgroup analysis of patients with preoperative stage III tumors showed a significantly improved survival in responders to chemotherapy compared to patients without response (p = 0.002) (Figure 3).

## Discussion

The poor prognosis of gastric carcinoma has remained unchanged for the past 2 decades with a 5-year-survival rate ranging between 40% and 50%. Surgery is the only curative treatment option for locally advanced disease. Furthermore, most patients are initially diagnosed at advanced tumor stages. Despite curative resection (R0), the overall recurrence rate is 69%. Locoregional relapse (87%) as well as lymphatic and peritoneal metastasis have a high likelihood [1,4,6]. Radiation and chemotherapy aim at controlling local tumor spread and eliminating disseminated tumor cells to prolong survival [18]. Intraoperative radiation succeeded in reducing locoregional relapse, but failed in improving overall survival [5]. The palliative implementation of PELF-CTx had a significant impact on the median survival [19]. Therefore, we investigated the influence of this protocol in the neoadjuvant setting on morbidity and mortality, tumor recurrence and prognosis.

Regarding size, our study ranges in the lower third of current literature [1,2,5,20]. With respect to age (median 69 years), lack of specific symptomatology in early tumor stages and typical symptoms in advanced stages of gastric cancer, diagnosis/staging and tumor localization, our study population compares well with similar trials [1,2,5,20]. Furthermore, our inclusion criteria are very similar to comparable studies [1,2,20]. In our study, patients with UICC stages Ib-III, diagnosed by imaging, were included. In comparable trials, only patients with stage IV and M + or only stages IIIa/b and IV were included [1,7,20].

Preoperative CTx aims at devitalizing and downsizing tumor tissue to increase the chance of curative resection [6]. In 20 patients (77%), there was an objective histological response. This is considerably more than in other studies where histological response was observed in 17% to 50% of cases [1,2,20-22]. An exceptional response was observed in cases of UICC stage III tumors (Table 2). In these patients a clear down-staging was recognizable [2,6,20]. Following CTx, the curative resection rate was 77%, thus exceeding reported R0-resection rates of around 60% [2,20]. However, compared to the result in patients who received surgical resection only (74%), we found no significant difference.

A comparison of pre- and postoperative tumor staging demonstrates a surprisingly low accuracy of implemented diagnostics. In the group without CTx, only 7 cases of 15 diagnosed stage III tumors were verified histologically. In the group with CTx, only 3 of 17 cases were confirmed histologically. This effect may however be explained by a down-staging of CTx. The similar high R0-resection rates in both study groups may be indicative of diagnostic imprecision, suggesting that more earlier tumor stages were in the CTx group. Unfortunately, explaining this in retrospect proves unfeasible.

Preoperative CTx is hoped to reduce recurrence rates (69%) by making curative resection more probable and by eliminating micrometastases [2]. In our study, the relapse rate was 38% after CTx versus 63% following surgery. Other studies report recurrence rates of 60% to 70% following CTx [5,20]. Especially local recurrence seems to be reduced after preoperative chemotherapy. 26% after surgery only and 12% of patients with CTx developed locoregional recurrence. These results are comparable with the local relapse after intraoperative radiation (10%) [5]. Recurrence in lymph nodes was only seen in patients without CTx and is explainable by the observed response especially of lymphatic micrometastases [20,22]. Nevertheless, recurrence in the peritoneum, 12% after CTx and 20% after surgery, and liver metastases couldn’t be prevented and were comparable with other trials [23].

First and foremost, neoadjuvant CTx aims prolonging overall survival [6]. Unfortunately, our results show that the overall survival is not improved under these conditions. The 5-year survival rate with chemotherapy was 38% versus 42% without. A comparable trial failed to show a benefit of chemotherapy as well [2,20]. Current trials hypothesize that an objective histological response is an important prognostic factor [2,6]. However, in our study responders had no better long-term outcome than patients without CTx (45% versus 42%) (Figure 2) [2,6,20]. The only exception to our observation was seen in a subgroup analysis of responders with stage III tumors. In this subgroup, the 5-year survival rate was 46% after CTx versus 31% after resection only (Figure 3).

## Conclusion

We regard advanced gastric cancer as a systemic disease. Meaningful prognostic criteria are intraperitoneal and local recurrence. Neoadjuvant CTx succeeds in reducing local relapse, but does not appear to impact the overall survival. Despite this lack of benefit as seen across all tumor stages, a subgroup of patients with stage III tumors seems to benefit significantly from neoadjuvant CTx. However, it remains challenging to select these patients due to the low accuracy of current diagnostics. In summary, additional therapeutic modalities, such as antibody treatment in combination with current standards of treatment will be necessary to improve the prognosis of advanced gastric cancer.

## Abbreviations

CTx: Chemotherapy; PELF: Cisplatin, Epirubicin, Leukovorin, 5-Fluoruracil; UICC: Union internationale contre le cancer; CR: Complete response; PR: Partial response; AJCC: American joint committee on cancer; JRSGC: Japanese research society for gastric cancer 1995.

## Competing interests

The authors declare that they have no competing interests.

## Authors’ contributions

RC was responsible for the data gathering and analysis of the data and also did the literature research. TO and RG did the surgery and RG also created the study design. GM and IG took care of the patients during chemotherapy and ensured the right application according to the PELF-protocol. MF performed the statistic analysis. All authors read and approved the final manuscript.

## Pre-publication history

The pre-publication history for this paper can be accessed here:

http://www.biomedcentral.com/1471-2482/14/5/prepub
